# Maintenance of multipotency of bone marrow mesenchymal stem cells on poly(ε-caprolactone) nanoneedle arrays through the enhancement of cell-cell interaction

**DOI:** 10.3389/fbioe.2022.1076345

**Published:** 2023-01-09

**Authors:** Xiaoxue Ren, Xiaoting Gao, Yicheng Cheng, Lingxia Xie, Liping Tong, Wei Li, Paul K. Chu, Huaiyu Wang

**Affiliations:** ^1^ Institute of Biomedicine and Biotechnology, Shenzhen Institute of Advanced Technology, Chinese Academy of Sciences, Shenzhen, China; ^2^ University of Chinese Academy of Sciences, Beijing, China; ^3^ Department of Stomatology, Jinling Hospital, School of Medicine, Nanjing University, Nanjing, China; ^4^ Department of Physics, Department of Materials Science and Engineering, Department of Biomedical Engineering, City University of Hong Kong, Kowloon, Hong Kong SAR, China

**Keywords:** autologous crystallization, bone marrow mesenchymal stem cells, multipotency, cell-cell interaction, poly (ε-caprolactone)

## Abstract

Mesenchymal stem cells (MSCs), with high self-renewal ability and multipotency, are commonly used as the seed cells for tissue engineering. However, the reduction and loss of multipotential ability after necessary expansion *in vitro* set up a heavy obstacle to the clinical application of MSCs. Here in this study, we exploit the autologous crystallization ability of biocompatible poly (ε-caprolactone) (PCL) to obtain uniformly distributed nanoneedle arrays. By controlling the molecular weight of PCL, nanoneedle with a width of 2 μm and height of 50 nm, 80 nm, and 100 nm can be successfully fabricated. After surface chemical modification with polydopamine (PDA), the water contact angle of the fabricated PCL nanoneedle arrays are reduced from 84° to almost 60° with no significant change of the nanostructure. All the fabricated substrates are cultured with bone marrow MSCs (BMMSCs), and the adhesion, spreading, proliferation ability and multipotency of cells on different substrates are investigated. Compared with the BMMSCs cultured on pure PCL nanoneedle arrays, the decoration of PDA can improve the adhesion and spreading of cells and further change them from aggregated distribution to laminar distribution. Nevertheless, the laminar distribution of cultured cells leads to a weak cell-cell interaction, and hence the multipotency of BMMSCs cultured on the PCL-PDA substrates is decimated. On the contrary, the pure PCL nanoneedle arrays can be used to maintain the multipotency of BMMSCs *via* clustered growth, and the PCL1 nanoneedle array with a height of 50 nm is more promising than the other 2 with regard to the highest proliferation rate and best multipotential differentiation ability of cultured cells. Interestingly, there is a positive correlation between the strength of cell-cell interaction and the multipotency of stem cells *in vitro*. In conclusion, we have successfully maintained the multipotency of BMMSCs by using the PCL nanoneedle arrays, especially the PCL1 nanoneedle array with a height of 50 nm, as the substrates for *in vitro* extension, and further revealed the importance of cell-cell interaction on the multipotency of MSCs. The study provides a theoretical basis for the behavioral regulation of MSCs, and is instructive to the design of tissue engineering scaffolds.

## 1 Introduction

Mesenchymal Stem Cells (MSCs) are commonly used as the seed cells for tissue engineering, which is due to their multidirectional differentiation potential, self-renewal ability and immunomodulatory function (Tavakoli et al., 2020; Chen et al., 2022). However, the scarcity of MSCs in human bodies is a great obstacle severely impeding the clinical application of MSCs. Take bone marrow mesenchymal stem cells (BMMSCs) as an example, BMMSCs only accounts for .1‰ of bone marrow cells, of which the activity would reduce rapidly with the increase of age (McKee and Chaudhry, 2017; Najar et al., 2021). Hence, the *in vitro* expansion of MSCs are necessary to complement this shortage. Conventional tissue culture polystyrene (TCP) is the most commonly-used substrate for *in vitro* MSCs expansion. Nevertheless, the *in vitro* expansion of MSCs using TCP will inevitably reduce the multipotency and self-renewal ability of MSCs, and ultimately causing MSCs useless for the clinical applications. Although a short-term maintenance of MSCs multipotency can be achieved by adding active molecules (e.g., endothelial growth factors, interleukin-6, immobilized leukemia inhibitory factor) to the culture medium, and maintaining an appropriate hypoxic environment for *in vitro* expansion (Grayson et al., 2007; Pricola et al., 2009; Khalil et al., 2020; Rawat et al., 2021; Samal et al., 2021). However, the related regulation processes are too complex and very hard to control. The recent studies on the *in vitro* regulation of MSCs behaviors have shown that the surface topography and biochemical properties of culture substrates can significantly influence the adhesion, proliferation, spread, distribution as well as the multipotency of MSCs (Li et al., 2017). Therefore, development of culture substrates that can help maintain the multipotential differentiation and self-renewal ability of MSCs during their *in vitro* expansion is urgently needed.

As one of the major properties of culture substrates, the surface topography of substrates is a crucial factor for determining the behaviors and fate of cultured cells (Chen et al., 2014; Cun and Hosta-Rigau, 2020). The methodologies for constructing micro/nanostructures on substrates are diversified, and studies have shown that the multipotency of MSCs can possibly be maintained by tuning the micro/nano-topography of substrates. For instance, (McMurray et al. (2011) combined electron beam lithography with hot embossing to fabricate PCL nanopillar arrays with 120 nm in diameter and 300 nm in space. By adjusting the arrangement of nanopillars, the improvement of osteogenic differentiation and the maintenance of MSCs multipotency were separately achieved. This study demonstrated the feasibility of maintaining MSCs multipotency *in vitro* by regulating the surface topography of culture substrates. In addition, Rico et al. (2016) fabricated a fibronectin nanonetwork by controlling the assembly of fibronectin and used it to maintain the multi-differentiation potency of MSCs. These studies demonstrated the importance of surface physical and chemical properties of substrates for maintaining the multipotency of cultured MSCs (Hofmeister et al., 2015). However, the aforementioned methods are still criticized for the operational complexity, which is unsuitable for further promotion. Therefore, it is of great research significance and clinical value to explore a substrate that is highly controllable, reproducible, simple to operate, easy to achieve large-scale preparation, and can effectively maintain the multipotency of MSCs during *in vitro* expansion.

It is worth noting that crystallization is a simple and controllable method for fabricating micro/nano-topographies, and more than two-thirds of the synthetic and natural polymers have crystallization ability (Crist and Schultz, 2016). Spherical crystals, string crystals, dendritic crystals, etc. with a wide range of dimensions can be obtained by regulating the mode and parameters of crystallization. What is more important, the methodology of crystallization also provides the advantages such as wide selection of materials, high reproducibility, simple operation as well as large-scale preparation, hence is drawing more and more attention in the field of materials regulating cellular behaviors and functions (Wang et al., 2012; Li et al., 2016; Ren et al., 2019).

Here in this study, we employed the biocompatible poly (ε-caprolactone) as the base material and utilized its semi-crystalline ability to fabricate nanoneedle arrays. By changing the average molecular weight of PCL, nanoneedles with average height of 50 nm, 80 nm and 100 nm were successfully obtained. In addition, the surface chemical state of PCL nanoneedles could be modified by polydopamine (PDA) without obvious changes in surface topography. Furthermore, the adhesion, spreading, distribution, proliferation, and multipotency of BMMSCs on different substrates were systematically studied and compared. Our study demonstrates that the nanoneedle of 50 nm high can be used to maintain the multipotency of MSCs during *in vitro* expansion and a positive correction between cell-cell interaction and multipotency of MSCs has been revealed. Overall, we have developed a simple, controllable, reproducible, and easy-to-achieve batch preparation method to maintain the multipotency of MSCs during *in vitro* expansion, which is also instructive for the understanding and *in vitro* regulation of stem cell behaviors and functions.

## 2 Materials and methods

### 2.1 Materials

Poly (ε-caprolactone) (PCL, M_n_∼10,000, 40,000, 80,000), dexamethasone, dimethyl sulfoxide (DMSO), Alizarin Red S, and Red Oil O were purchased from Sigma-Aldrich (United States). Dopamine hydrochloride, Triton X-100, isobutylmethylxanthine (IBMX), indomethacin, insulin, Tris and bovine serum albumin (BSA) were purchased from Aladdin (China). All chemical reagents were used as received. Sprague Dawley (SD) rats of 4 weeks were obtained from Guangdong Charles River Laboratory Animal Technology Co., Ltd (China). Fetal bovine serum (FBS, Gibco, United States), α-MEM culture medium (Hyclone, United States), .25% trypsin (Gibco, United States), penicillin/streptomycin (P/S, Gibco, United States), PBS (pH 7.4, Gibco, United States), 4% paraformaldehyde (Beyotime Biotechnology, China), cell counting kit-8 (CCK-8, Beyotime Biotechnology, China), Alexa Fluor™ 488 Phalloidin (Life Technologies, United States), Hoechst 33,258 (Life Technologies, United States), Trizol (life technologies, United States), anti-Nanog antibody (Proteintech, United States), anti-N-cadherin antibody (Proteintech, United States), Alexa Fluor 488-conjugated AffiniPure Goat Anti-Rabbit IgG (H + L) (Proteintech, United States), Alexa Fluor 561-conjugated AffiniPure Goat Anti-Rabbit IgG (H + L) (Proteintech, United States), PrimeScript™ RT Master Mix Kit (TaKaRa, Japan) and TransStart Top Green qPCR SuperMix (TransGen Biotech, China) were applied according to the instructions.

### 2.2 Preparation of PCL nanoneedle arrays

The isotropic films were prepared by controlling the self-crystallizing process of poly (ε-caprolactone) (PCL). In particular, 30 μl of .02 g/ml PCL solution in trichloromethane was dropped on a clean mica sheet (1 cm^2^ × 1 cm^2^) and dried at room temperature for 1 h. Afterwards, the formed PCL films were treated at 80°C for 15 min to eliminate the heat history. Finally, the PCL films were quickly transferred into liquid nitrogen in an open system and maintained overnight for full self-crystallization.

### 2.3 Decoration of PDA (PCL-PDA)

PCL samples were placed vertically in a 24-well plate. Dopamine was dissolved in 10 mM Tris-HCl (pH 8.5) by magnetic stirring to .02 g/ml (Lee et al., 2007). In the next step, the solution was quickly added to the 24-well plate with an amount of 4 ml per well and then reacted for 1 h in the dark. The samples were finally washed with sufficient deionized water and dried at room temperature.

### 2.4 Surface characterization

An X-ray photoelectron spectrometer (EscaLab 250Xi, Thermal Fisher, United States) was applied to analyze the surface chemical states of PCL and PCL-PDA. Compared to PCL, the appearance of N-signal peak at ∼400 eV of PCL-PDA is the hallmark of successful modification. Atomic force microscope (AFM, MultiMode 8, Bruker, United States) was applied to characterize the micro/nanostructures of PCL and PCL-PDA films in the tapping mode in the air. The obtained data were analyzed using the Nanoscope software. Furthermore, the hydrophilicity of the films in the air were measured using a contact angle meter (Data-Physics OCA-20 Appa-raus, Germany). 4 μl of the deionized water were dropped onto different sample surfaces, and the water contact angles were measured after stabilization for 5 s.

### 2.5 Isolation and culture of BMMSCs

BMMSCs were obtained from 4-weeks SD rats using the whole bone marrow adherence method as previously reported (Friedenstein et al., 1970; Li et al., 2015). Briefly, rats were firstly sacrificed with cervical dislocation, and the femurs and tibias were flushed with basal α-MEM medium containing 10% fetal bovine serum and 1% penicillin/streptomycin to obtain the bone marrow. Afterwards, the bone marrow was cultured in a cell culture incubator (37°C, 5% CO_2_) for 36 h and then rinsed with phosphate buffer solution (PBS). The fresh culture medium was added and refreshed every 2 days. Finally, when being fused to 90%, the cells were digested with .25% trypsin and passaged 1:3. BMMSCs at passages 1–3 were utilized in our study.

### 2.6 Cell adhesion, proliferation, spreading, and distribution

Taking TCPS as control, the adhesion and proliferation of BMMSCs on PCL and PCL-PDA were determined by CCK-8 assay. Briefly, the various samples were placed in a 24-well plate and sterilized by UV irradiation. BMMSCs seeded onto different samples at a density of 2 × 10^4^/cm^2^ and the culture medium was changed every 2 days. After incubation for 1 day, 3 days, 7 days, and 10 days, CCK-8 reagent was added to the basal α-MEM medium at a ratio of 1:10 (v/v), and then added to the culture wells for another 1 h of incubation. Finally, the absorption of each group at 450 nm was measured by a microplate reader (BL340, Biotech, United States).

Fluorescent staining was utilized to characterize the morphology and distribution of cells on different samples. In detail, BMMSCs were cultured on the PCL and PCL-PDA for 1 day and 7 days, fixed with 4% paraformaldehyde and then successively stained with Alexa Fluor™ 488 Phalloidin and Hoechst 33,258. Subsequently, the stained cells were observed under a confocal laser scan microscope (A1, Nikon, Japan) at 408 nm and 488 nm.

### 2.7 Immunofluorescent staining for multipotency and cell-cell interaction

After 14 days of incubation, BMMSCs on different samples were fixed with 4% polyparaformaldehyde for 15 min. After permeation with .1% Triton X-100 for 5 min, the cells were blocked with 10% normal goat serum, separately incubated with the primary anti-Nanog antibody (1:200) and primary anti-N-cadherin antibody (1:400) for 2 h at 37°C, and then the second antibody Alexa Fluor 561-conjugated AffiniPure Goat Anti-Rabbit IgG (H + L) (1:500) and Alexa Fluor 488-conjugated AffiniPure Goat Anti-Rabbit IgG (H + L) (1:500) were correspondingly added into each well for another 1 h of incubation at 37°C. Finally, the nuclei of BMMSCs were stained with Hoechst 33,258, and cells were observed under a confocal laser scan microscope (A1, Nikon, Japan) at 408 nm, 488 nm, and 561 nm.

### 2.8 Induced differentiation and staining

BMMSCs were cultured on different samples for 14 days. Afterwards, the cells were resuspended and seeded onto the 24-well plates at a density of 2 × 10^4^/cm^2^. When being fused to 80%, the cells were further subjected to osteogenic induction by using the basic culture medium added with 10 mM β-glycerophosphate, 50 μM ascorbic acid, and .1 μM dexamethasone (Wu et al., 2021; Xie et al., 2021). After 14 days of osteogenic induction, the cells were fixed with 4% paraformaldehyde and then stained with Alizarin Red S solution (.1% w/v) for 30 min. Adipogenic induction of BMMSCs was implemented by adding 1 μM dexamethasone, 10 mg/L insulin, .5 mM isobutyl-1-methylxanthine and .2 mM indomethacin to the basic medium for cell culture (Sekiya et al., 2004). After 7 days of adipogenic induction, the cells were fixed and stained with Red Oil O solution (6 mg/ml in the mixture of isopropoyl alcohol and H_2_O (3:2, v/v)) for 30 min. Finally, all the stained samples were observed by light microscopy (IX71, Olympus, Japan).

### 2.9 Quantitative real time-polymerase chain reaction (RT-PCR)

To investigate the multipotency and induced differentiation, BMMSCs were cultured on different samples for 14 days, and then total RNA was extracted using the Trizol reagent according to the manufacturer’s protocol. Reverse transcription was performed using Life ECO TC-96 (BIOER, China) and quantitative RT-PCR was performed on the CFX 96 RT-PCR system (Bio-Rad, United States) using a mixture of SYBR Green Realtime PCR Master Mix, cDNA in each group, as well as forward and reverse primers listed in Table 1. The expression of various genes was normalized to the housekeeping gene glyceraldehyde-3-phosphate dehydrogenase (GAPDH).

**TABLE 1:** Primers used for quantitative RT-PCR.

Gene	Forward primer (5′–3′)	Reverse primer (5′–3′)
GAPDH	GTT​ACC​AGG​GCT​GCC​TTC​TC	GAT​GGT​GAT​GGG​TTT​CCC​GT
OCN	TGG​CAC​CAC​CGT​TTA​GGG​CA	TTT​GGA​GCA​GCT​GTG​CCG​TC
OPN	AAC​CGC​ACC​CAC​AAC​CGA​GT	ACC​GTG​TTT​CGC​TCT​GGG​GT
LPL	ATG​GCA​CAG​TGG​CTG​AAA​GT	CCG​GCT​TTC​ACT​CGG​ATC​TT
C/EBPα	GAC​CAT​CCG​CCT​TGT​GTG​TA	CTG​ACA​TTG​CAC​AAG​GCA​CC
Nanog	CCG​TGT​TGG​CTG​CAT​TTG​TC	ACC​TGG​GGG​AGG​ATA​GAG​TG
Sox-2	AGA​ACT​AGA​CTC​CGG​GCG​AT	ACC​CAG​CAA​GAA​CCC​TTT​CC
N-Cadherin	GTA​TGG​ATG​AAA​CGC​CGG​GA	TTG​TGG​CTC​AGC​GTG​GAT​AG

### 2.10 Statistical analysis

All the biological experiments were performed at least in triplicate (*n* ≥ 3) and each biological experiment was repeated at least three times (*N* ≥ 3). The results were presented as mean ± standard deviation. The normality test was performed with SPSS software and the Student’s *t*-test was performed to determine the levels of statistical significance between different groups. * Denotes *p* < .05, ** denotes *p* < .01 and *** denotes *p* < .001. A difference of *p* < .05 was considered to be significant and that of *p* < .01 was considered to be highly significant.

## 3 Results and discussion

### 3.1 Preparation of PCL nanoneedle arrays and PDA decoration

Straight-chain PCL, which is a synthetic polymer with excellent biocompatibility, is utilized in this study. PCL has been approved by Food and Drug Administration and widely used in tissue engineering (Woodruff and Hutmacher, 2010). As a typical semi-crystalline polymer, PCL can form regular spherical crystalline structures with radii up to 500 microns by autologous crystallization and can be used to obtain a variety of specific microstructure by epitaxial crystallization (Wittmann and Paul, 1991; Toda et al., 2012). Therefore, PCL nanoneedle microcrystal arrays with uniform distribution can be fabricated by controlling the process of autologous crystallization.

To our knowledge, the molecular weight of polymer, crystallization mode and crystallization temperature are decisive to the crystallization process and hence can influence the fabricated structures (Zhang et al., 2016). Generally speaking, the higher the molecular weight is, the longer the polymer chains are, and the longer duration it will take to achieve regular alignment (Wurm et al., 2012). In this regard, three different kinds of PCL samples with number average molecular weight of 10,000 (PCL1), 40,000 (PCL4) and 80,000 (CPL8) respectively are chosen for the following study.

In addition, the higher the crystallization temperature is, the faster the polymer chains move and the shorter time it needs to form crystals (Phillipson et al., 2015). Since the glass transition temperature of PCL is −60°C, the crystallization rate of PCL at room temperature is too fast to form spherical crystals or ring-banded spherical crystals of large size eventually. Hence, crystallization temperature of PCL should be decreased to slow down its crystallization rate. As shown in schematic diagram 1, the PCL films were firstly obtained by the drop addition method, then the thermal history was eliminated by heat treatment at 80°C (20°C above the melting point of PCL) for 15 min. To slow down the crystallization rate of PCL as much as possible, the PCL films were rapidly transferred to liquid nitrogen (−186°C) atmosphere, in which the ambient temperature would gradually increase with the volatilization of liquid nitrogen.

**FIGURE 1:**
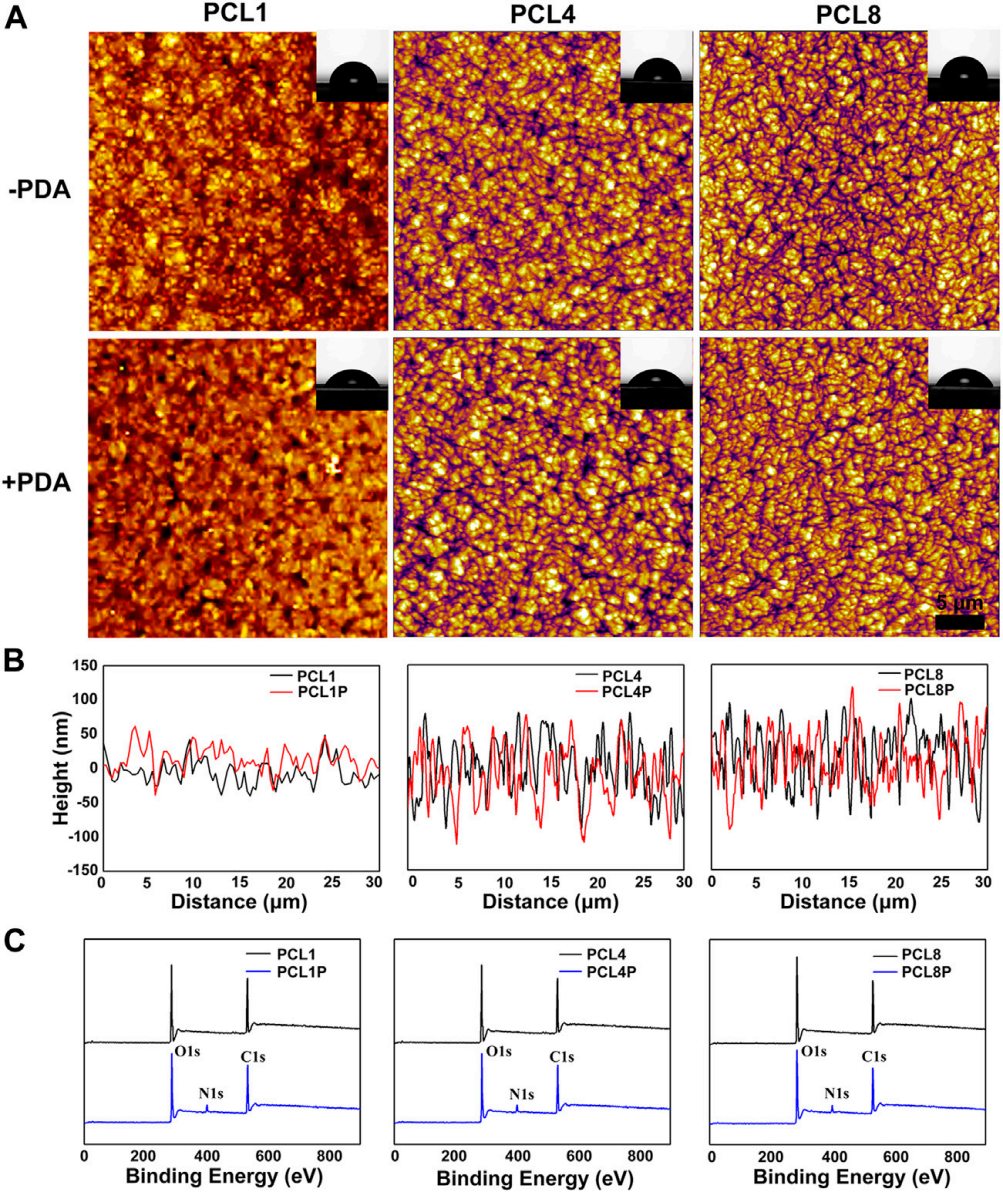
Characterization of nanoneedle arrays. **(A)** AFM images of nanoneedle arrays, the inset images show the corresponding water contact angles. **(B)** The height of nanoneedle arrays. **(C)** Survey XPS spectra determined on different samples.

As shown in Figure 1A, nanoneedle arrays of PCL could be successfully fabricated according to our protocol, and the width of nanoneedles did not change significantly with the increase of molecular weight. On the other hand, the average height of nanoneedles could be increased from 50 nm to 80 nm or even 100 nm by changing the number average molecular weight of PCL from 10,000 to 80,000 (Figure 1B). This is probably because the increase of molecular weight and the growth of molecular chains may lead to the increase in molecular chain folding space, so the height of crystalline nanoneedles increases. In the meantime, there is no significant difference in the width of crystalline nanoneedles because the nucleation rate of PCL with different molecular weight is similar.

In addition to the construction of micro/nanostructures, surface chemical modification was further performed in this study using a classical strategy of dopamine polymerization. In particular, a large number of catechol groups could be introduced onto the nanoneedle arrays of PCL by the rapid and spontaneous polymerization of dopamine under alkaline and aerobic conditions (Liu et al., 2014). Consequently, the introduced catechol groups could significantly improve the surface hydrophilicity of PCL substrates (Lee et al., 2007). As shown in Figure 1C, the appearance of the characteristic peak of N element at ∼400 eV indicated the successful chemical modification of PCL *via* dopamine polymerization. Furthermore, the water contact angle of PCL1, PCL4, and PCL8 are 83.48° ± 1.37°, 84.17° ± .77°, and 87.49° ± 1.52°, which could be respectively reduced to 59.49° ± .55° (PCL1-PDA), 62.81° ± .33° (PCL4-PDA) and 65.29° ± 1.20° (PCL8-PDA) after PDA decoration. At the same time, the thin coatings of polydopamine did not significantly change the topography of nanoneedle arrays (Figure 1A). Therefore, we could successfully fabricate nanoneedle crystalline arrays by regulating the crystallization process of PCL, with the height of crystalline nanoneedles ranging from 50 nm to 100 nm. The surface chemical states of nanoneedle arrays were also tunable by subsequent modification with PDA, which could avoid the obvious changes in surface topography.

### 3.2 Adhesion, spreading, distribution, and proliferation ability of BMMSCs on different substrates

With the rapid development of material biology, numerous studies have revealed that the behaviors and functions of stem cells can be readily regulated by the chemical and topographic features of culture substrates through contact guidance effect (Nguyen et al., 2016). Therefore, the adhesion, spreading, distribution and proliferation of BMMSCs on different substrates were systematically investigated.

**FIGURE 2:**
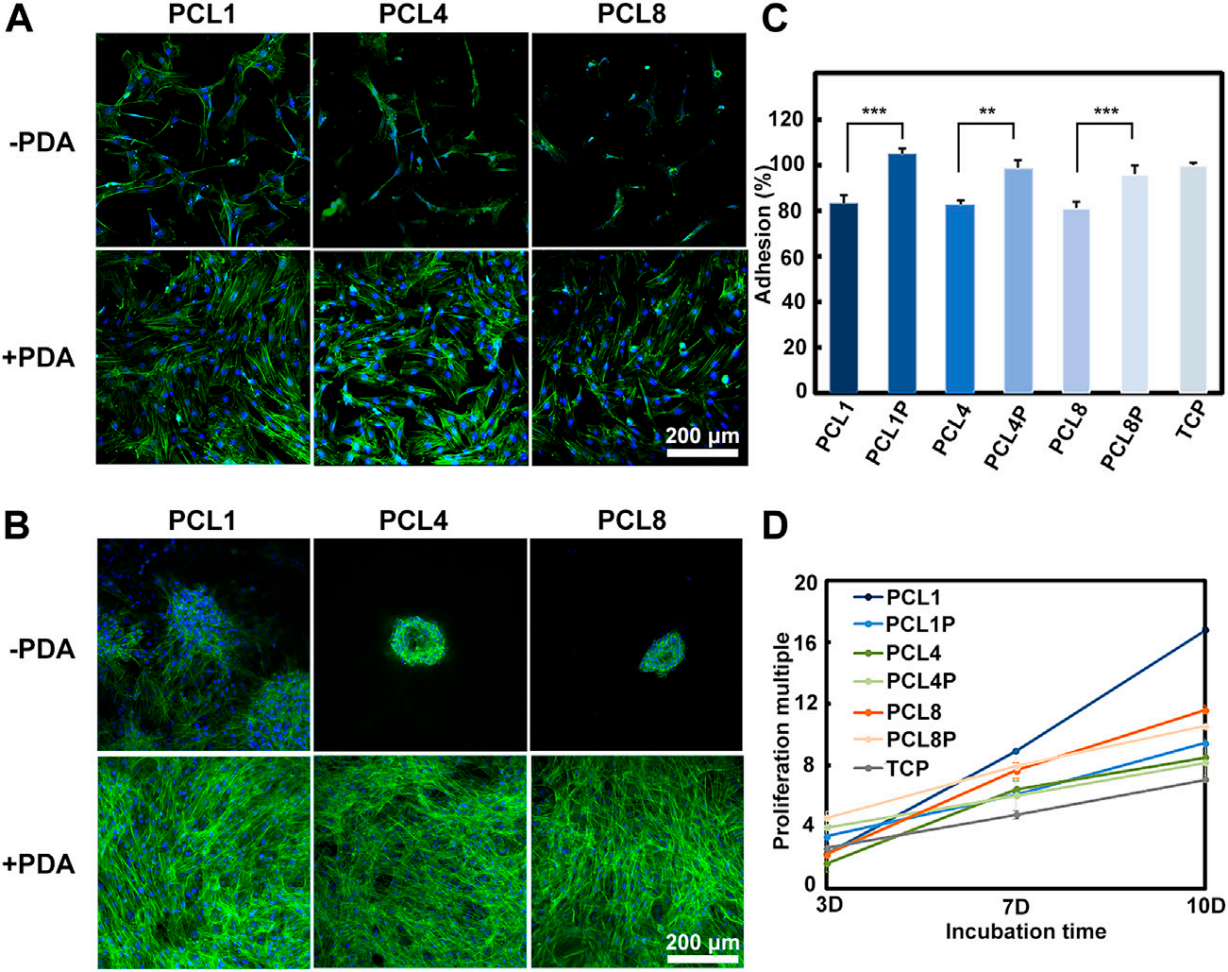
Behaviors of BMMSCs cultured on different substrates. **(A)** The fluorescent images of BMMSCs cultured on different substrates for 1 day and **(B)** 7 days (green: Actin; blue: Nuclear) (*n* = 3). **(C)** CCK-8 assay of BMMSCs cultured on samples for 1 day (*n* = 4). **(D)** The proliferation of MSCs on different substrates for up to 10 days (*n* = 4).

It is well recognized that the primary adhesion and subsequent spreading are the initial two steps of cells in contact with substrates, which depends on the physicochemical-biological properties of the substrates (Martino et al., 2018). As shown in Figures 2A, C, BMMSCs could adhere on different substrates after 24 h of cell culture, but the cell number and spreading area were different. The adhesion number of BMMSCs in the pure PCL groups (PCL1, PCL4, PCL8) was about 80% of that on TCP, and the spreading area decreased with an increase of the height of nanoneedle arrays. In contrast, the adhesion number and spreading area of BMMSCs in the PCL + PDA groups (PCL1P, PCL4P, PCL8P) were comparable to that of TCP. This is because PDA is abundant with the catechol groups, which can increase the surface hydrophilicity of PCL substrates (Liu et al., 2014). Moreover, the introduction of polar groups such as hydroxyl groups after PDA modification can increase the adsorption of proteins from culture medium onto the substrates, which is beneficial to cell adhesion spreading (Liu et al., 2016).

With the increase of culture time to 7 days, as shown in Figure 2B, agglomerative growth of BMMSCs could be observed in the pure PCL groups, which was accompanied by reduced actin expression and a decrease in BMMSCs size. Additionally, the agglomerative size decreased with the increase of nanoneedle height. In contrast, the cells cultured on the PDA-modified substrates showed similar laminar growth as those cultured on TCP, and there were no significant changes with the increase of nanoneedle height. Our results demonstrated that the hydrophilicity of substrates is crucial for the different growth modes of stem cells in long-term culture.

The proliferation ability is the basic function requirement of MSCs when they are used for tissue engineering. However, the telomeres length of MSCs would reduce during the traditional culture and passage *in vitro*, causing the decrease and even loss of proliferation ability (Trivanovic et al., 2015). So it is important to investigate whether our substrates could maintain the proliferation activity of MSCs *in vitro* or not. As shown in Figure 2D, with the increase of cell culture time, BMMSCs could proliferate on all substrates. Noteworthily, the proliferation rate of BMMSCs on PCL1 was significantly higher than on the other substrates, indicating that this substrate was the most favorable for the proliferation of BMMSCs *in vitro*.

Taken together, we could regulate the adhesion, spreading, distribution and proliferation behaviors of BMMSCs by changing the topographical and chemical properties of culture substrates. A three-dimensional clustered growth of BMMSCs could be achieve on the pure PCL substrates, which was similar to the three-dimensional culture of cells in matrigel. Most importantly, our results demonstrated that the nanoneedle arrays with a height of 50 nm obtained from PCL1 were more preferable than the others for facilitating the proliferation of BMMSCs *in vitro*.

### 3.3 Multipotency of BMMSCs on different substrates

In our research, BMMSCs cultured on the pure PCL nanoneedle arrays also grew in clustered aggregates, which was similar to the three-dimensional culture of cells in three-dimensional scaffolds and commercial Matrigel (Tavassoli et al., 2018). Hence, we further investigated the multipotency of BMMSCs cultured on PCL nanoneedle arrays and compared them with the PCL-PDA groups.

**FIGURE 3:**
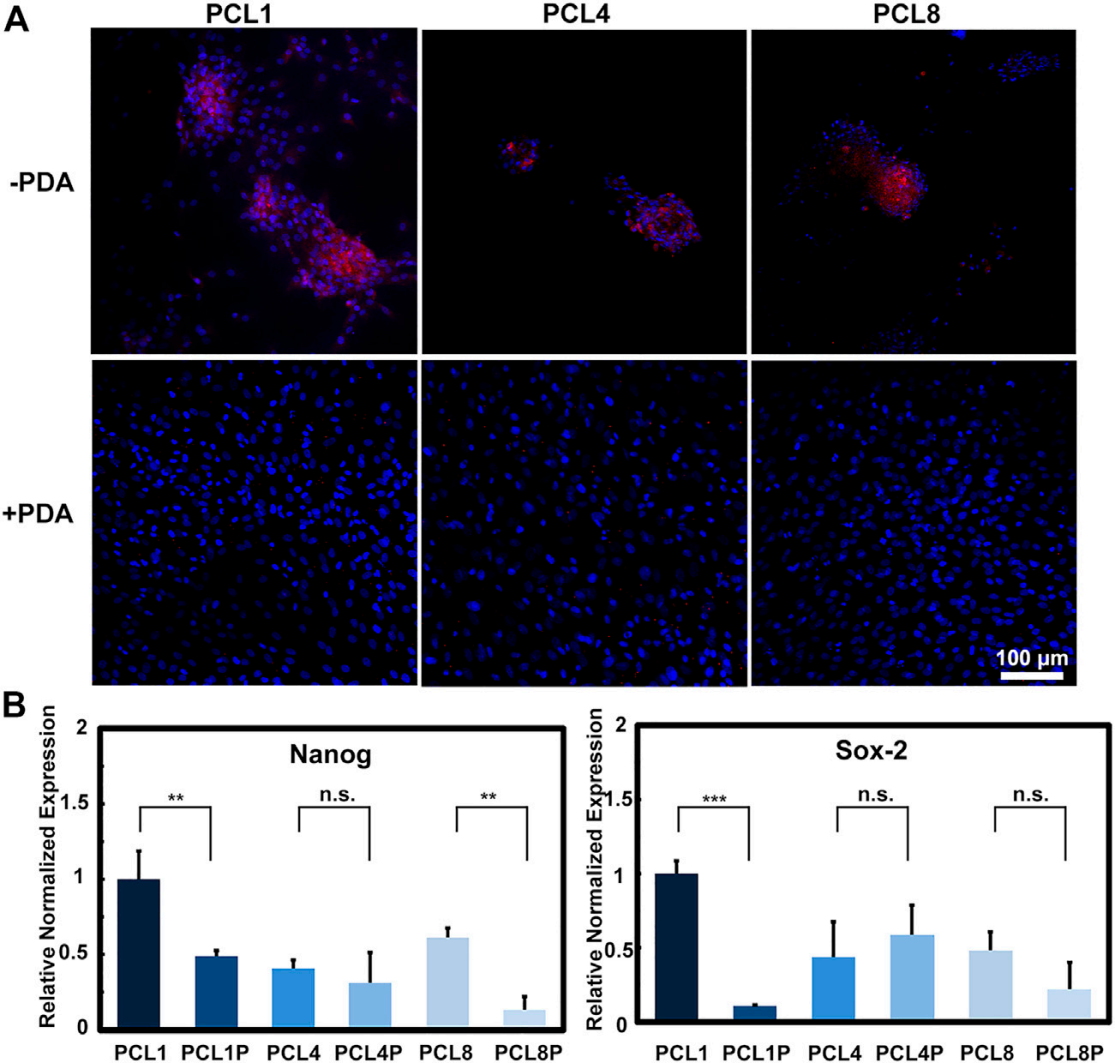
The multipotency of BMMSCs after being cultured on different substrates for 14 days. **(A)** Immunofluorescence staining of Nanog protein (red: Nanog, blue: Nuclear) (*n* = 3) and **(B)** gene expressions of Nanog and Sox-2 of the cultured cells (*n* = 4).

As is well known, Nanog and Sox-2 are pluripotent transcription factors, which can be used to evaluate the differentiation potentials of stem cells including BMMSCs (Hoch and Leach, 2014; Yoon et al., 2014). Therefore, BMMSCs were cultured on PCL and PCL-PDA for 14 days, and then examined for the expression of Nanog protein using immunofluorescence staining and quantitatively analyzed for the gene expression of Nanog and Sox-2 using quantitative RT-PCR. As shown in Figure 3A, clustered cells could be observed on the pure PCL nanoneedle arrays, which showed relatively higher expression of Nanog protein than the laminar distributed cells cultured on PCL-PDA. Figure 3B revealed that the gene expressions of Nanog and Sox-2 showed a similar trend as the Nanog protein levels detected in different groups, of which the BMMSCs cultured on the pure PCL nanoneedle array of 50 nm high (PCL1) showed the highest expressions of multipotential markers.

Multipotency also refers to the capacity of stem cells differentiating into particular cell types. As for BMMSCs, the capability of osteogenic and adipogenic differentiation are representative of the multipotency (Jiang et al., 2002). Hence, we further analyzed the induced differentiation ability of BMMSCs cultured on different substrates. After being cultured on PCL and PCL-PDA for 14 days with a basal culture medium, the cells were resuspended into a 24-well plate and induced with the osteogenic culture medium and adipogenic culture medium respectively.

**FIGURE 4:**
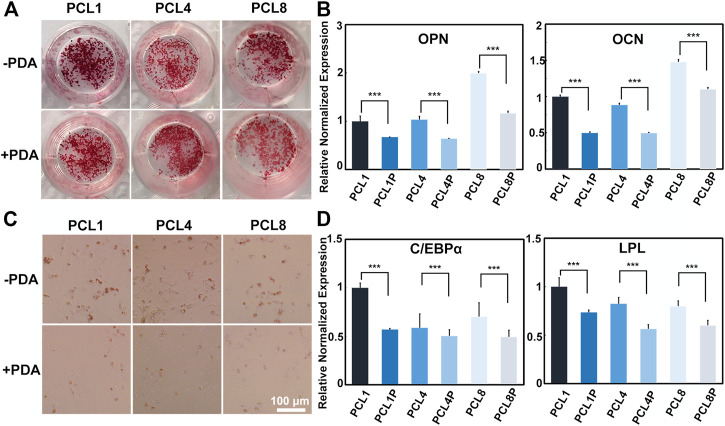
Induced differentiation of BMMSCs after being cultured on different substrates. **(A)** Alizarin Red S staining and **(B)** osteogenesis-related gene expressions of the cells after osteogenic induction for 14 days; **(C)** Red Oil O staining and **(D)** adipogenesis-related gene expressions of the cells after adipogenic induction for 7 days (*n* = 4).

After osteogenic induction for 14 days, the osteogenic differentiation of BMMSCs was determined by Alizarin Red S staining and quantitative RT-PCR. During the process of osteogenic differentiation, BMMSCs would secrete calcium and deposit it around the cells to form calcified nodules, and the amount and size of calcified nodules increase with the maturation of osteoblasts. As shown in Figure 4A, the number and size of calcified nodules formed by cells harvested from the pure PCL nanoneedle arrays were larger than those detected in the PCL-PDA groups, indicating that the clustered cells grown on PCL provided better capacity of osteogenic differentiation. By comparing PCL nanoneedle arrays with different heights, we found that the cells harvested from the nanoneedle array with a height of 50 nm showed the best capacity of *in vitro* mineralization. At the same time point, quantitative RT-PCR was also performed to analyze the gene expressions of osteopontin (OPN) and osteocalcin (OCN) as they are typical biomarkers of osteogenic differentiation (Tong et al., 2019; Mo et al., 2021; Chen et al., 2022). As shown in Figure 4B, the cells cultured on the pure PCL nanoneedle arrays exhibited the higher expression of osteogenesis-related gene than the PCL-PDA groups. Both the Alizarin Red S staining and quantitative RT-PCR results showed that the BMMSCs cultured on all groups, especially the pure PCL nanoneedle array with a height of 50 nm (PCL1) and 100 nm (PCL8), provided relatively high capacity of osteogenic differentiation.

As for adipogenic differentiation, the resuspended cells were incubated with the adipogenic culture medium for 7 days, and then determined by Oil Red O staining and quantitative RT-PCR. By inducing the differentiation of stem cells to adipocytes, lipid droplets would form, increase and then fuse to larger-sized droplets with the development of adipocyte maturity (Ntambi and Kim, 2000). As shown in Figure 4C, the lipid droplets formed by cells harvested from the pure PCL nanoneedle arrays were more mature than those detected in the PCL-PDA groups. Further analysis by quantitative RT-PCR in Figure 4D showed that the adipogenesis-related gene expressions of cells harvested from the pure PCL nanoneedle arrays were higher than their PDA-modified counterparts, indicating that the cells expanded on PCL substrates provided the better capability of adipogenic differentiation than those in PCL-PDA groups. Noteworthily, the cells harvested from the pure PCL nanoneedle array with a height of 50 nm (PCL1) exhibited the highest expression of CCAAT/enhancer binding proteins α (C/EBPα) and LipoProtein Lipase (LPL). It means that this kind of PCL nanoneedle array is likely better than others for maintaining the adipogenic differentiation ability of cultured cells.

The above results showed that the pure PCL nanoneedle arrays were more favorable than their PDA-modified counterparts for maintaining the multipotency of BMMSCs during *in vitro* expansion. Especially, BMMSCs harvested from the pure PCL nanoneedle array with a height of 50 nm (PCL1) could provide the best performances of multidirectional differentiation.

### 3.4 Cell-cell interaction of BMMSCs on different substrates

**FIGURE 5:**
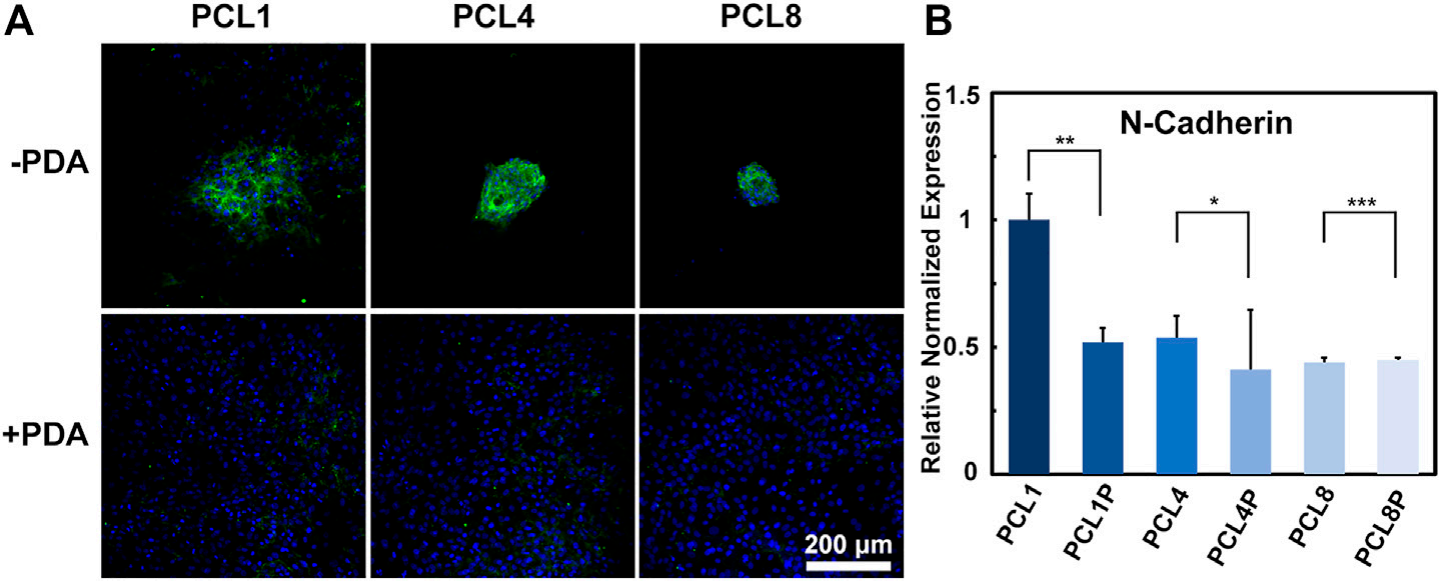
Cell-cell interaction of BMMSCs cultured on different substrates for 14 days. **(A)** Immunofluorescence staining for N-Cadherin protein (green: N-Cadherin; blue: Nuclear) (*n* = 3) and **(B)** gene expressions of N-Cadherin of the cultured cells (*n* = 4).

**SCHEME 1:**
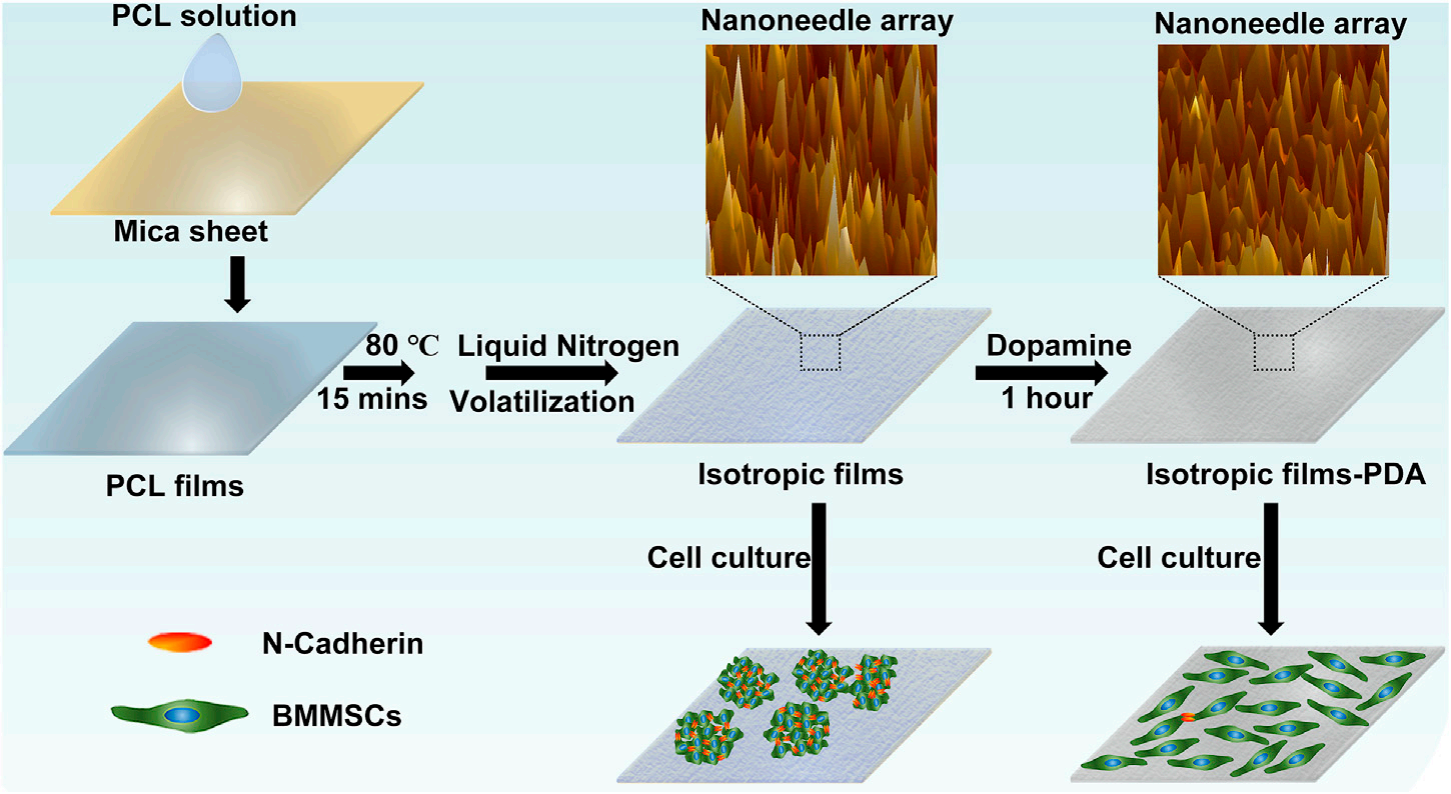
The process of sample preparation and cell culture.

On one hand, cells can sense the extracellular matrix through integrin etc. and achieve a response to the extracellular matrix through cell-substrate interactions. On the other hand, cells can also interact with each other by direct communication with neighboring cells through desmosomes, gap junctions and tight junctions (Belardi et al., 2020). In the present study, the fabricated pure PCL nanoneedle arrays could achieve aggregated growth of the cultured BMMSCs, but the further modification of PCL nanoneedle arrays with PDA resulted in a typical laminar distribution of cultured cells, eventually compromising the multipotency of stem cells during *in vitro* expansion. Probably, the changes in cell functions might be related to cell-cell interaction. Hence, after culturing BMMSCs on different samples for 14 days, we further investigated the expression of N-cadherin (a specific marker of cell-cell adhesion) by immunofluorescence staining and quantitative RT-PCR (Cosgrove et al., 2016). As shown in Figure 5A, the expression of N-Cadherin was significantly higher in agglomerated-grown BMMSCs of the pure PCL groups than that in laminar-grown cells of the PCL-PDA groups. Further combined with the quantitative RT-PCR results shown in Figure 5B, we found that the pure PCL nanoneedle array with a height of 50 nm (PCL1), could not only support the highest proliferation ability while maintaining the multipotency of cultured cells, but also provide the strongest cell-cell interactions among the different substrates Scheme 1.

## 4 Conclusion

In summary, our study demonstrates a feasible strategy for the *in vitro* expansion of MSCs without compromising their multipotency. In particular, PCL nanoneedle arrays can be fabricated by regulating temperature of autologous crystallization process, and utilized as the substrates for the *in vitro* expansion of MSCs. Furthermore, the height of PCL nanoneedle arrays is tunable, and the PCL1 nanoneedle array of 50 nm high is more promising than the others with regard to the highest proliferation rate and best multipotential differentiation ability of cultured cells. Although the further decoration of PDA can improve the adhesion and spreading of cultured cells, it also changes the cells from aggregated distribution to laminar distribution, which will weaken the strength of cell-cell interaction and compromises the multipotency of cells eventually. Our study not only broadens the application of polymer crystals, but also provides a theoretical basis and experimental guidance for the *in vitro* regulation of MSCs, and makes the tissue engineering using multipotential MSCs more feasible.

## Data Availability

The original contributions presented in the study are included in the article/supplementary material, further inquiries can be directed to the corresponding authors.
